# The Impact of a Simulated Intrauterine Device Insertion Clinic on Osteopathic Medical Students’ Knowledge, Attitude, and Self-Efficacy Regarding Intrauterine Devices

**DOI:** 10.7759/cureus.84642

**Published:** 2025-05-22

**Authors:** Alexandria L Betit, Lucy P Kelly, Rahul Garg, Praful G Patel

**Affiliations:** 1 Obstetrics and Gynecology, Alabama College of Osteopathic Medicine, Dothan, USA; 2 Obstetrics and Gynecology, Southeast Health Medical Center, Dothan, USA

**Keywords:** contraception, intrauterine device, intrauterine simulation, long-acting reversible contraceptives (larc), medical school curricula, medical students, osteopathic, reproductive health, simulation training, women’s health

## Abstract

Background

Despite high rates of unintended pregnancy in the United States, there is a lack of comprehensive, high-quality education about intrauterine devices (IUDs) within U.S. medical school curricula. This study evaluated the impact of a simulated IUD insertion clinic on knowledge, attitudes, self-efficacy, and comfort regarding IUDs among preclinical osteopathic medical students in the southern United States.

Methods

A pre- and post-intervention survey was administered to preclinical medical students attending an IUD insertion simulation clinic. The survey assessed students' knowledge of IUDs, attitudes towards the role of different healthcare providers in contraceptive counseling and placement, perceived self-efficacy in counseling and IUD placement, and personal comfort with IUD use. McNemar tests were used to conduct the bivariate analysis of change in responses before and after the clinic.

Results

A total of 57 students completed the surveys before and after the clinic. The clinic significantly improved students' knowledge regarding the IUD mechanism of action, return of fertility, and common misconceptions (p<0.05). Further, the participants were more likely to recommend IUDs for appropriate candidates under 19 years of age (p<0.01) but less likely to recommend them for patients with active chlamydial infections (p<0.05) after the clinic. Attitudes toward IUD placement by midwives and nurse practitioners became significantly more favorable after the clinic (p<0.001). Additionally, participants reported increased comfort with personally receiving or recommending IUDs (p<0.05) and demonstrated a notable increase in perceived self-efficacy for IUD counseling and supervised placement (p<0.0001).

Conclusion

A short IUD simulation clinic effectively enhanced the knowledge, attitudes, comfort, and self-efficacy of osteopathic medical students. The study findings support the inclusion of hands-on IUD training in medical school curricula to better prepare future physicians for comprehensive contraceptive counseling and care. Furthermore, increased recognition of the role of advanced practice providers in reproductive healthcare may improve access to long-acting reversible contraceptives, particularly in underserved communities. Implementing similar simulation experiences across medical education could further strengthen reproductive health competencies and address barriers to contraceptive access.

## Introduction

In 2019, unintended pregnancies accounted for 41.6% of all pregnancies in the United States, with one-third occurring among women in their 20s [1,2]. This percentage remains significantly higher as compared to other Western nations [2]. Limited access to contraception, inadequate sexual and reproductive health education, and socioeconomic disparities remain significant barriers to reducing high rates of unintended pregnancy [2].

Despite these barriers, a wide range of contraceptive methods are available for women, with options for individual preference, efficacy, route of administration, duration of action, and potential adverse effects. Reversible contraceptive options include behavioral methods (e.g., coitus interruptus, the rhythm method, abstinence), barrier methods (e.g., cervical cap, condoms, vaginal diaphragm, spermicidal agents, contraceptive gels), short-acting hormonal methods (e.g., oral contraceptive pills (OCPs), transdermal patches, vaginal rings), and long-acting methods (e.g., intramuscular injections such as depot medroxyprogesterone acetate, intrauterine devices (IUDs), and subdermal implants) [3]. Among the available contraceptive options, long-active reversible contraceptives (LARCs), including the subdermal implant and the IUD, are the most effective, with failure rates of less than 1% per year [3].

IUDs specifically have gained increasing popularity, with usage rising 6.2% annually from 2006 to 2017 [4]. However, widespread misconceptions and misinformation persist, potentially limiting further adoption among women [5]. Misinformation is not limited to patients but also extends to clinicians, which may influence their likelihood of recommending IUDs [6,7]. A few prior initiatives aimed to enhance medical students’ experiences with IUDs by implementing insertion clinics designed to improve their knowledge, attitudes, confidence, and comfort with IUDs [8,9]. The results suggest that incorporating such training into medical curricula may enhance the utilization of IUDs in clinical practice [8,9].

We found limited research on IUD insertion clinics at osteopathic medical schools, which tend to graduate a higher proportion of primary care physicians than allopathic institutions [10]. Osteopathic medical graduates play a critical role in providing safe and effective contraceptive methods to diverse patient populations. Further, osteopathic medical students in the southern United States may have different baseline knowledge and attitudes regarding IUDs than allopathic medical students in other states. This study involved implementation of an IUD insertion clinic for osteopathic preclinical medical students in the southern United States (Alabama). The primary objective was to evaluate changes in knowledge, attitudes, self-efficacy, and comfort related to IUDs, thereby assessing the potential benefits of simulation-based training in an osteopathic medical education setting.

Preliminary findings from this study were disseminated through two poster presentations titled "Impact of an IUD Insertion Clinic on Medical Students’ Knowledge, Attitude, and Perceived Self-Efficacy Regarding IUDs." These were presented at the American College of Osteopathic Family Physicians (ACOFP) Virtual 62nd Annual Convention on February 22, 2024, and at the American College of Osteopathic Obstetricians and Gynecologists (ACOOG) 91st Annual Conference on May 6, 2024, where the study received second place in a national poster competition. Additionally, an abstract of this study was published in the North American Proceedings in Gynecology and Obstetrics (NAPGO) as part of the ACOOG conference proceedings.

## Materials and methods

Data and sample

An educational IUD clinic was developed and implemented in a single session during the 2022-23 academic year at an osteopathic medical school in Alabama, United States. The Institutional Review Board at the Alabama College of Osteopathic Medicine approved this study under the Exempt category one protocol number HS230109-E. The participants included preclinical medical students. Eligibility criteria included being over the age of 18 and having attended middle and high school within the United States. We recruited participants via an online sign-up sheet distributed to the school-issued email accounts of eligible medical students. The email included the event description and emphasized that participation in both the clinic and research study was anonymous and voluntary. The study conducted surveys before and after the clinic to examine the changes in knowledge, attitude, self-efficacy, and comfortability with IUDs. Of the approximately 400 medical students who received the email, 57 attended and participated in the clinic. To encourage attendance, food was provided; however, no monetary compensation was offered. Completion of the pre-and post-surveys was considered to imply informed consent.

Measures

The educational IUD clinic was designed based on the methodologies of two previous studies that were conducted among 137 preclinical and 35 clinical medical students located in the Pacific Northwest (Washington) and Northeast (Massachusetts), respectively [8,9]. The clinic included a single session comprising a 30-minute lecture delivered by a board-certified obstetrician and gynecologist (OBGYN), a brief IUD insertion demonstrated by the same lecturer, and a 30-minute hands-on session where participants practiced IUD insertion using uterine models under supervision.

**Figure 1:**
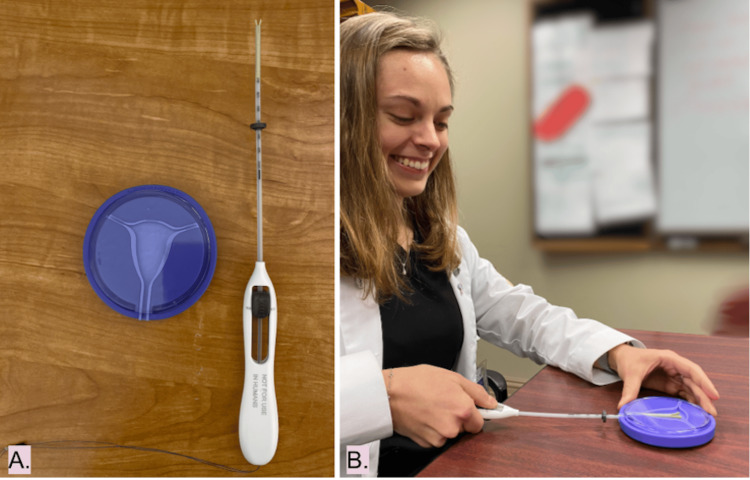
(A,B) Placebo IUD kits utilized in the educational intrauterine device (IUD) clinic. Kits included plastic discs in the shape of a uterus and IUD applicator (A) both of which were used during the insertion portion of the demonstration and for student-assisted participant practice (B). The individual shown in the image is the author, who has provided informed consent for publication of this image.

The lecture, presented using PowerPoint, provided an overview of LARCs with a focus on IUDs. The topics covered included historical background, indications, complications, mechanisms of action, effectiveness, benefits, duration of use, timing of insertion, required medications for insertion, and common misconceptions. Demonstration IUD insertion kits, which included a uterine-shaped plastic model and an IUD applicator, were provided by Medical Students for Choice (MSFC) (Figure 1 A,B). Throughout the session, medical student researchers assisted participants in the insertion process. To ensure each participant had sufficient hands-on practice, no more than three medical students were assigned to each uterine model.

To assess the effectiveness of the intervention, the participants completed a paper survey both before and after the didactic presentation and simulation experience (see Appendix). The pre- and post-training surveys were matched by a unique identifier, keeping the results anonymous. The pre- and post-surveys included questions related to: (1) students’ desired medical specialty, (2) participants' main source of information regarding IUDs, (3) number of IUDs the participants have seen placed in the past, (4) personal experiences with IUDs, (5) baseline IUD knowledge including common misconceptions, (6) attitudes regarding IUDs, and (7) personal comfortability and self-efficacy regarding IUD counseling and placement. The pre-survey had questions regarding age, sex, ethnicity, marital status, and parental status to assess the demographic information. Additionally, the pre-survey included questions about the state of residence, geographic environment (rural vs. urban), and average household income before the age of 18. Geographic environment was self-reported by participants based upon the 2010 Census urban-rural definition where urban areas contain populations of 2,500 or more people and rural areas contain populations of 2,500 or less people [11]. In total, there were 53 survey items listed on the pre-survey, including the demographic questions. The post-survey asked the same 38 questions from the pre-survey on knowledge, attitudes, personal comfortability, and self-efficacy regarding IUDs. Many of the survey questions were modified with author permission from a prior study of medical students’ knowledge of and comfort with IUDs [8].

Analysis

The survey responses were linked between the pre-and post-surveys using unique identifier codes. For the statistical analysis, we combined the responses of ‘Strongly agree’ and ‘Agree’ into ‘Agree’ and ‘Strongly Disagree’ and ‘Disagree’ into ‘Disagree’ to increase the cell sample sizes. We calculated the percentages of responses for each survey question reported in the tables. We used the McNemar parametric tests to measure the changes in categorical survey responses regarding knowledge, attitudes, personal comfortability, and self-efficacy before and after the training clinic. We used SAS software 9.4 (SAS Institute, Cary, NC, USA) for all the statistical analysis, with p-values less than 0.05 considered statistically significant.

## Results

**Figure 2:**
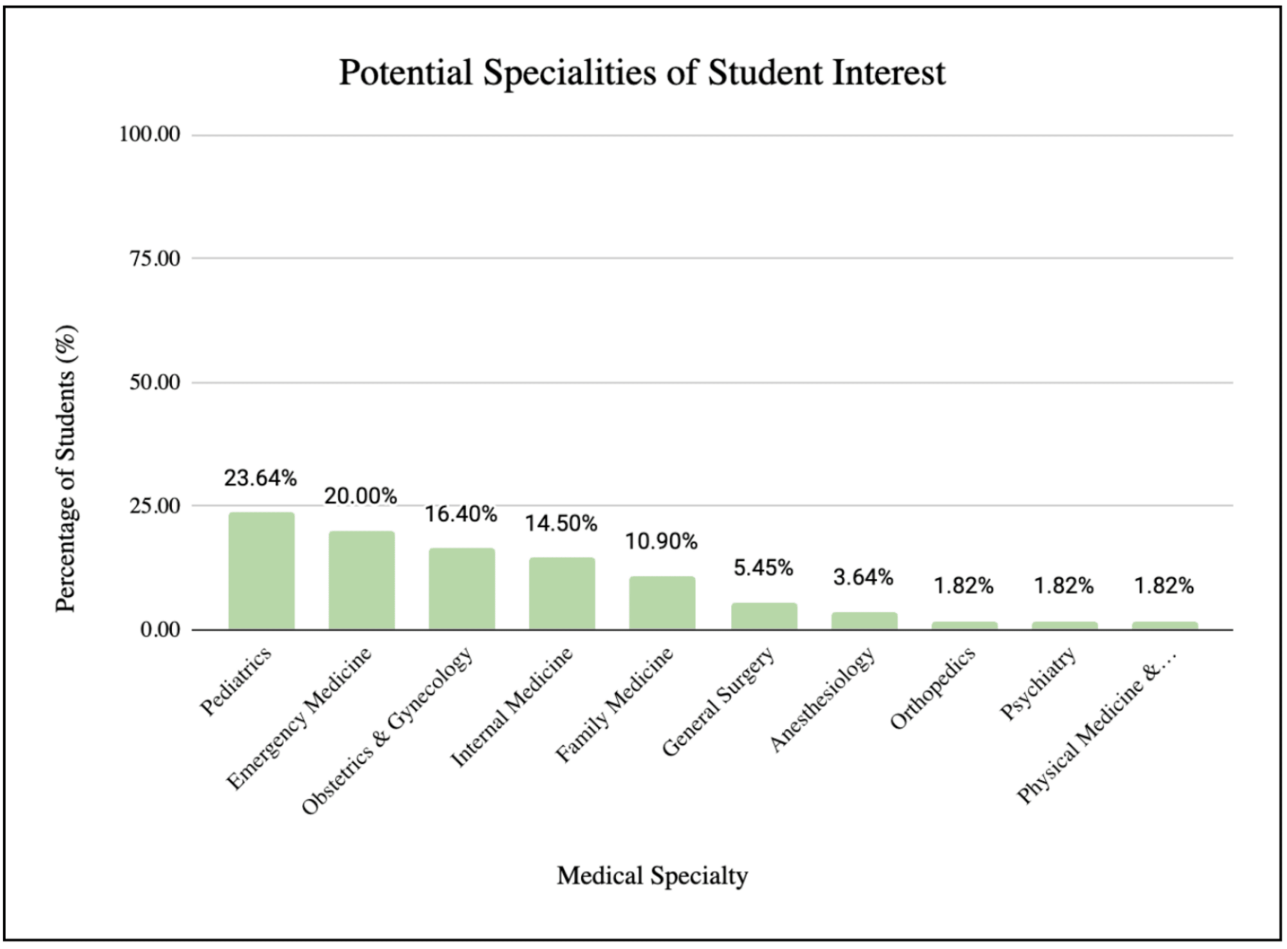
Participants’ intended medical specialty of choice after graduation.

A total of 57 preclinical medical students participated in the IUD insertion clinic and completed the pre-and post-surveys. Three students’ responses could not be linked due to missing identifier codes and hence, 54 responses were included in the repeated before and after bivariate analysis. Among the 57 respondents, 47% were first-year and 53% were second-year osteopathic medical students. The participants reported residing throughout all four regions of the United States within both urban and rural areas with 67% residing in the Southern United States and 91% within an urban area. Most participants identified as single (88%), Caucasian (72%) females aged 26 years (range: 22-34 years) who reported an average household income of $50,000 to $100,000 (27%) before the age of 18. Participants were interested in pursuing a wide variety of medical specialties at the time of the study (Figure 2). Primary care specialties were the most commonly selected residency choices among study participants, with pediatrics representing a leading preference at 23.64% (Figure 2).

The majority of participants indicated that they obtained most of the information about IUDs from a healthcare provider (45.61%) while the fewest participants reported receiving such information from family or a medical school lecture (7.02%). Additionally, a large proportion of participants had never observed an IUD insertion in a clinical setting (87.72%). Most participants had no personal experience with IUDs (61.40%); however, the majority (94.74%) reported knowing at least one friend or family member who had an IUD.

Students demonstrated a significant increase in the majority of IUD-related knowledge questions following the IUD insertion clinic (Table 1). For example, students showed higher knowledge in understanding IUD efficacy and return of fertility, as well as in dispelling common misconceptions (Table 1). Additionally, after the clinic, most participants indicated they would recommend an IUD for an otherwise appropriate candidate under 19 years old (p<0.01), but were less likely to recommend one for a patient with an active chlamydial infection (p<0.05) (Table 1).

**Table 1:** Impact of the intrauterine device (IUD) clinic on knowledge regarding IUDs. *p-values were not calculated for some questions due to low or zero sub-category sample (cell) sizes. p-values < 0.05 were considered statistically significant.

Survey Item (Sample size = 54)	Correct Before Clinic	Correct After Clinic	Chi-square value	p-value
If 100 women used any type of IUD for 1 year, how many would have an unintended pregnancy?	70.37%	94.44%	8.89	0.003
How quickly does fertility typically return after any type of IUD is removed?	37.04%	90.74%	29.00	<0.0001
What is the primary mechanism of action of a levonorgestrel IUD in contraception?	31.48%	29.63%	0.06	0.808
What is the primary mechanism of action of a copper IUD in contraception?	56.60%	88.68%	-	*
A patient must have previously given birth to be eligible for an IUD.	96.30%	94.44%	0.20	0.655
IUD insertion is painful.	85.19%	87.04%	0.14	0.705
IUDs can cause ectopic pregnancies.	35.19%	100.0%	-	*
IUDs negatively interfere with sexual pleasure.	94.44%	100.0%	-	*
IUDs can cause weight gain.	59.26%	77.78%	4.12	0.041
A patient must obtain a pap smear before receiving an IUD.	25.93%	48.15%	6.55	0.011
IUD is likely to cause the following:				
Pelvic inflammatory disease (PID)	64.81%	98.25%	-	*
Spontaneous abortion (miscarriage)	64.81%	100.0%	-	*
Get misplaced/fall out	68.52%	72.22%	2.50	0.475
Assuming she is an otherwise good candidate, I would recommend an IUD for a patient:				
Who has never been pregnant	81.48%	98.15%	-	*
Who is under 19 years old	64.81%	90.74%	14.40	0.002
Who currently has chlamydia	64.81%	87.04%	10.62	0.014
Who has had more than one vaginal delivery	64.81%	98.15%	-	*

Following the insertion clinic, there was a significant increase in the number of students who supported internal medicine physicians, pediatricians, midwives, and nurse practitioners (NPs) as qualified providers for IUD placement (Table 2). Additionally, independent of clinic participation, most students agreed that all listed clinician types should be prepared to counsel patients on IUDs (Table 2). Participants also demonstrated increased comfort with IUDs after the clinic, with more students indicating they would recommend an IUD to a family member (p<0.05) (Table 3). Finally, students showed a significant increase in confidence regarding both patient counseling and supervised IUD placement following the clinic (Table 4).

**Table 2:** Impact of the IUD clinic on attitudes regarding IUD placement and counseling by various clinicians. *p-values were not calculated for some questions due to low or zero sub-category sample (cell) sizes. p-values < 0.05 were considered statistically significant.

Survey Item (Sample size=54)	Strongly Agree/Agree Before the Clinic	Strongly Agree/Agree After the Clinic	Chi-square values	p-value
The following clinicians should be able to place IUDs:				
Obstetrician-Gynecologists	98.11%	100.0%	-	*
Family Medicine Physicians	68.52%	98.15%	-	*
Internal Medicine Physicians	61.11%	96.30%	20.00	0.0002
Pediatricians	53.70%	96.30%	24.00	<0.0001
Midwives and Nurse Practitioners	62.26%	96.23%		0.0003
The following clinicians should be able to counsel patients about IUDs:				
Obstetrician-Gynecologists	100.0%	100.0%	-	*
Family Medicine Physicians	100.0%	100.0%	-	*
Internal Medicine Physicians	88.89%	98.15%	3.57	0.059
Pediatricians	88.89%	100.0%	-	*
Midwives and Nurse Practitioners	94.44%	96.30%	-	*

**Table 3:** Impact of the IUD clinic on personal comfortability. *p-values were not calculated for some questions due to low or zero sub-category sample (cell) sizes. p-values < 0.05 were considered statistically significant.

Survey Item (Sample size = 54)	Strongly Agree/Agree Before the Clinic	Strongly Agree/Agree After the Clinic	Chi-square values	p-value
I am interested in learning more about IUDs	100%	100%	-	*
I want to learn how to place IUDs	100%	100%	-	*
Contraceptive counseling will be a part of my practice	79.25%	98.11%	-	*
I would recommend an IUD to my family member	72.22%	92.59%	11.00	0.012
I would get an IUD placed or I would recommend an IUD to my partner	65.38%	76.92%	4.49	0.214

**Table 4:** Impact of the IUD clinic on perceived self-efficacy. *p-values were not calculated for some questions due to low or zero sub-category sample (cell) sizes. p-values < 0.05 were considered statistically significant.

Survey Item (Sample size=54)	Strongly Agree/Agree Before the Clinic	Strongly/Agree After the Clinic	Chi-square values	p-value
I feel confident that I am able to counsel patient about the IUD	16.67%	94.44%	44.00	< .0001
I know the steps to place an IUD	9.26%	98.15%	-	*
I feel comfortable placing an IUD independently in a plastic model	7.41%	90.74%	-	*
I could teach another student how to place an IUD in a plastic model	3.77%	94.34%	-	*
I feel comfortable placing an IUD in a patient under faculty supervision	7.41%	75.93%	45.00	< .0001

## Discussion

Our study examined the impact of an IUD insertion clinic on knowledge, attitudes, self-efficacy, and comfort related to IUDs, specifically among osteopathic medical students in the southern United States. The intervention resulted in significant improvements in knowledge, attitudes, and perceived self-efficacy regarding IUDs. Misinformation regarding IUDs is prevalent among both patients and healthcare providers [5]. Common misconceptions include concerns about pain during insertion and continued use, as well as beliefs that IUDs exacerbate acne [5]. Other widespread myths include that IUDs cause abortion, pelvic inflammatory disease (PID), infertility, ectopic pregnancy, weight gain, hair loss, osteoporosis, and cancer [5]. A literature review analyzing a 2008 survey of 816 healthcare providers revealed that over half did not consider adolescents, nulliparous individuals, immediate postpartum patients, or those with a history of PID or ectopic pregnancy to be appropriate candidates for IUDs [5]. Furthermore, inconsistent and inaccurate knowledge of contraceptive methods among healthcare providers may limit their ability to offer comprehensive contraceptive counseling, ultimately hindering efforts to reduce unintended pregnancies [12]. Evidence suggests that when adolescents receive accurate information about LARCs, they are more likely to choose LARC methods [5]. Therefore, clinicians play a crucial role in increasing the utilization of LARCs, particularly among adolescents, a population in which 82% of pregnancies are unintended [5].

This inconsistent and inaccurate knowledge surrounding IUDs emphasizes the need for improvements in medical education regarding contraception, particularly IUDs [12]. A few of our surveyed participants indicated they received most of the information about IUDs from a medical school lecture, but most had never observed an IUD insertion in a clinical setting. A literature review assessing family planning curricula at 20 U.S. medical schools between 2016 and 2019 found that instruction predominantly focused on OCPs and LARCs, primarily delivered through PowerPoint lectures or class handouts [13]. Contraceptive education within medical schools varies significantly in content and depth, often being brief and challenging to assess [13]. Additionally, some medical schools do not provide formal education on family planning at all [14]. In response, some students have sought supplemental training through elective courses during their fourth year of medical school to better prepare for providing comprehensive reproductive healthcare [14].

Notably, students exhibited significantly more favorable attitudes towards IUD placement by midwives and NPs following the clinic. This shift highlights a potential gap in medical education curricula regarding the role of advanced practice providers (APPs) in women’s healthcare within the United States, APPs frequently serve as the first point of contact for women in medically underserved areas [15,16]. Specifically, NPs and midwives are more likely to provide holistic care and have been shown to improve patient-provider communication [17,18]. Given these potential contributions to comprehensive reproductive healthcare, increasing awareness among medical students regarding the essential role of APPs may enhance the accessibility and utilization of LARCs, including IUDs, in historically underserved populations.

Prior studies targeting both preclinical and clinical allopathic medical students in the Northwest (Washington) and Northeast (Massachusetts) have conducted similar IUD simulation interventions, demonstrating improvements in knowledge, comfort, and perceived self-efficacy following participation [8,9]. Our study, Bartz et al. and Field et al. all demonstrate increased IUD knowledge and perceived self-efficacy post-intervention [8,9]. Furthermore, all participants in Field et al.’s study reported that the simulation was valuable [9], reinforcing the effectiveness of simulation-based learning in medical education.

Neither Field et al. nor Bartz et al. specifically addressed changes in participants’ attitudes regarding which clinicians, including midwives and nurse practitioners, should be able to perform IUD placement or provide contraceptive counseling [8,9]. This distinction highlights an area where the present study contributes novel insights by evaluating shifts in medical students' perceptions of the role of APPs in reproductive healthcare. These findings, along with those of the present study, underscore the effectiveness of IUD simulation training and the need for its broader integration into medical education curricula to enhance knowledge, confidence, attitudes, and clinical competency in contraceptive care.

Furthermore, neither of the aforementioned studies included participants from osteopathic institutions [8,9]. Comprehensive education on IUDs is particularly crucial in osteopathic medical schools, as a significant proportion of osteopathic graduates pursue careers in primary care [10]. Notably, most students who participated in this simulation expressed interest in primary care specialties. Primary care providers, including pediatricians and family medicine physicians, have extensive exposure to diverse patient populations, including adolescents, positioning them to play a critical role in expanding access to IUDs [6,19]. Pediatricians, in particular, are uniquely positioned to provide contraception counseling and services to adolescents - a population disproportionately affected by unintended pregnancies [19]. However, without adequate training in comprehensive contraceptive care, these providers may lack the confidence and competency necessary to effectively offer IUDs, leaving significant gaps in adolescent reproductive healthcare [19].

In addition, primary care providers serve as essential healthcare access points in rural areas, where OBGYNs may be scarce [20]. Despite their potential to improve contraceptive access, many family medicine physicians and pediatricians report insufficient training pertaining to IUDs, which hinders their ability to offer this method to patients [6,19]. A national probability survey of family medicine physicians found that although many did not currently offer LARCs, they expressed interest in additional training to enhance their competency in providing these methods [6]. The survey concluded that hands-on training opportunities could significantly increase future patient access to IUDs [6].

Research has shown that medical students often remain in the region where they complete their training [21]. This simulation was completed at a medical institution in the southeastern United States, a region characterized by lower contraceptive utilization rates and higher unintended teen birth rates [22,23]. A literature review found that Alabama had the lowest LARC utilization rate in the country, with only 6.1% of individuals using this form of contraception [23]. Given these regional disparities, integrating simulation-based training into medical education programs may enhance future clinicians’ ability to provide IUDs, thereby increasing access, improving utilization, and potentially reducing unintended pregnancies in underserved areas. These findings align with prior studies, which suggest that simulation-based training effectively enhances student confidence in intrauterine procedures [24,25].

Based on the findings of this study, as well as previous simulated training interventions [8,9], we propose that incorporating an IUD insertion clinic into the curriculum could better prepare students as they transition to residency, particularly given the significant variation in reproductive health education across medical schools. According to Casas et al., successful programs integrating LARCs into their internal medicine residency education primarily occurred with a small number of residents in a high-volume setting [26]. While these settings enabled a limited number of residents to gain proficiency in LARC procedures, broader educational models offering more elective opportunities and external clinical experiences ensured exposure for all residents [26]. This approach of providing a more flexible learning opportunity for medical students could serve as an additional point of exposure before they graduate.

When implementing IUD insertion clinics and additional LARC education within the medical school curriculum, it is essential to consider learner outcomes, available resources, and flexible learning opportunities [26]. During the preclinical years, IUD insertion clinics could be paired with corresponding lectures and conducted in small-group simulation settings [9]. Additionally, an evening session or after-class session could be offered for students interested in specialties involving reproductive care [9]. Simulation should be an essential part of the preclinical curriculum and not solely limited to the clinical years of medical school [9]. With multiple learning opportunities, students can begin building their foundation in women’s health early on in their medical education [9]. Schools could also offer a similar IUD workshop for fourth-year medical students interested in going into a field that provides reproductive health care [9]. Before entering residency training, this type of hands-on workshop could integrate their knowledge and skills in a meaningful way [9].

Although LARCs are one of the most effective and most frequently mentioned forms of contraception in medical school curricula, they remain the fourth most common contraceptive method after OCPs, sterilization, and condoms [13]. Our study demonstrated that students who participated in the clinic felt more confident in IUD counseling and supervised placement. By fostering this confidence, medical schools can empower future physicians to promote LARCs as a safe and effective contraceptive option.

Due to the replicative nature of this study, several limitations align with those observed in previous research [8,9]. Our study was conducted on a small osteopathic medical school campus and may not be generalizable to all training sites. Furthermore, our small sample size - comprising osteopathic medical students who may have had a stronger interest in women’s health - limits the generalizability of our findings. We believe that this limited representation among participants may have contributed to the absence of significant changes in attitudes between pre- and post-survey responses. However, this study contributes to the existing literature on IUD simulation interventions, complementing previous studies conducted at allopathic institutions in different regions of the United States [8,9]. While generalizability may be a concern in single-institution studies, the consistency of findings across multiple settings suggests that the impact of such interventions is not limited to a specific geographic location or medical education model. In this study, the workshop was offered only once, making it difficult to predict how this clinic will impact the future practice of the participants. Future studies could implement this clinic in multiple sessions over a longitudinal timeline, including follow-up surveys with the same participants. However, maintaining contact with graduates after they lose access to their medical school email could pose a challenge. Finally, the PowerPoint presentation shown after the pre-survey and before IUD simulated practice could have been more comprehensive in covering all survey content. Instead, we relied heavily on the presenter to verbally convey key educational points. Future studies could address this by implementing standardized PowerPoint guidelines to ensure all essential IUD information is consistently covered.

The primary strengths of this study include the high response rate among participants who attended the simulation clinic, which was likely facilitated by the use of paper surveys administered immediately following the session. This method minimized the risk of nonresponse bias, as participants were more inclined to complete the survey in person rather than disregarding or overlooking an emailed post-survey. Additionally, our sample consisted of preclinical medical students with diverse specialty interests, contributing to the generalizability of our findings. Furthermore, the use of donated IUD kits reduced the financial burden of the workshop, making it a cost-effective and easily replicable educational intervention.

Future research aims include expanding the IUD insertion clinic from a single-day event to multiple sessions conducted over a longitudinal timeline to achieve broader generalizability. Implementing workshops at key educational milestones - such as the end of preclinical years, the end of fourth-year medical school, and during the intern year of residency - would allow for the assessment of sustained changes in physician knowledge, attitudes, comfort, and self-efficacy regarding IUD insertion. Additionally, this expanded model would facilitate a more in-depth examination of how demographic factors, including socioeconomic status, geographic location, and gender, influence the workshop's impact on student learning. Future clinics should also incorporate content on contraceptive coercion to ensure trainees are equipped to provide patient-centered, ethical contraceptive counseling that respects individual autonomy.

To further assess the clinic's effectiveness and adaptability, other medical schools could implement similar workshops across diverse geographic areas, enabling a comparative analysis of potential confounding variables. Future iterations may also broaden the scope of the workshop to include hands-on demonstrations of other LARCs, such as a subdermal implant insertion, providing participants with a more comprehensive contraceptive education.

## Conclusions

This study highlights the value of simulation-based training in improving medical students’ knowledge, attitudes, and confidence regarding IUDs. Given that many osteopathic students pursue primary care, ensuring they receive adequate contraceptive education is essential, particularly in regions where access to reproductive healthcare is limited. Our findings underscore existing gaps in medical curricula, as most participants had not received significant IUD-related instruction through medical school lectures or observed an insertion in a clinical setting. The study also sheds light on the role of APPs in expanding contraceptive access. After participating in the clinic, students expressed significantly more favorable attitudes toward IUD placement by midwives and nurse practitioners, suggesting that increased exposure to their contributions to reproductive healthcare could foster stronger collaborative care models and improve patient access to long-acting contraception.

By adding to the existing literature on IUD simulation training, this study builds on previous research conducted at allopathic institutions in different regions of the United States. Future efforts should explore implementing similar educational interventions across diverse medical schools to assess their effectiveness in various settings. Expanding hands-on contraceptive training in medical curricula - especially in osteopathic programs in the South - could better prepare future physicians to counsel patients and perform IUD insertions confidently. Ultimately, integrating these training opportunities may help improve access to long-acting contraception and reduce unintended pregnancies, particularly in underserved communities.

## Appendices

Pre-Training Intrauterine Contraception Questionnaire

Please provide the last 4 digits of your cell phone (or home phone) number. _____________. This is used to pair your pre- and post- survey responses. It is not used to identify you in any way.

1. Please circle your year in medical school: 

FIRST YEAR (Class of 2026)            SECOND YEAR (Class of 2025)     (Please circle one)

2. What is your current age: ______ years

3. What is your sex?

    a. Female

    b. Male

    c. Other: ________________

4. Which of the following best describes you? (please choose one answer)

    a. Asian or Pacific Islander

    b. Black or African American

    c. Native American or Alaskan Native

    d. White or Caucasian

    e. Multiracial or Biracial

    f. Prefer not to answer

5. Are you of Hispanic or Latino origin?

    a. Yes

    b. No

6.     Has the entirety of your middle school and high school education been in the United States of America?

    a. Yes

    b. No

IF YOU ANSWERED NO TO THE ABOVE QUESTION, PLEASE DO NOT COMPLETE THE REMAINING QUESTIONS ON THIS SURVEY, THANK YOU!

7. Which state did you grow up in (majority of time before age 18)? Circle one.

Alabama     Alaska          Arizona      Arkansas        California 

Colorado    Connecticut     Delaware      Florida    Georgia 

Hawaii     Idaho       Illinois     Indiana   Iowa

Kansas     Kentucky       Louisiana     Maine   Maryland

Massachusetts     Michigan     Minnesota     Mississippi    Missouri

Montana       Nebraska      Nevada      New Hampshire

New Jersey      New Mexico     New York     North Carolina

North Dakota   Ohio     Oklahoma     Oregon     Pennsylvania

Rhode Island        South Carolina       South Dakota    Tennessee   Texas

Utah    Vermont       Virginia    Washington

West Virginia   Wisconsin   Wyoming

8. What geographic environment did you grow up in (majority of time before age 18)?

    a. Urban (defined as areas with populations of 2,500 or more)

    b. Rural (defined as areas with populations of 2,500 or less)

9. What was your average household income before age 18?

    a. Below $10k

    b. $10k-$50k

    c. $50k-$100k

    d. $100k-$150k

    e. $150k-$200k

    f. Over $200k

10. What is your marital status?

    a. Married

    b. Single

    c. Prefer not to say

11. Do you have children?

    a. Yes

    b. No

    c. Prefer not to say

12. If you had to choose a specialty today, which one would you be most likely to pick? (Please circle one)

    a. Family Medicine

    b. Pediatrics

    c. OB-GYN

    d. Internal Medicine

    e. Emergency Medicine

    f. Other: ___________

13. What is your main source of information about IUDs? (Please circle one)

    a. Medical school lecture

    b. Friends

    c. Family

    d. Healthcare provider

    e. Other: ____________

14. How many IUD placement procedures have you seen in a clinical setting?  (Place one check mark)

    a. ___ (0)

    b. ___ (1-2)

    c. ___ (3-4)

    d. ___ (>5)

15. The following statements have to do with your personal experiences with IUDs. (Circle YES or NO)

    I or my partner has an IUD, or have had one in the past.         

                YES                    NO

    I know of at least one friend or family member that has ever had an IUD 

                YES                    NO

16. If 100 women used any type of IUD for 1 year, how many would have an unintended pregnancy?

    a. <1%

    b. 1-2%

    c. 3-5%

    d. >5%

17. How quickly does fertility typically return after any type of IUD is removed?

    a. Within 1 month

    b. 1-2 months

    c. 3-6 months

    d. 6-12 months

18. What is the primary mechanism of action of a levonorgestrel IUD in contraception?

    a. preventing fertilization

    b. preventing implantation

    c. disrupting an implanted embryo

19. What is the primary mechanism of action of a copper IUD in contraception?

    a. preventing fertilization

    b. preventing implantation

    c. disrupting an implanted embryo

*Correct answers are underlined above.

20. How likely is an IUD to cause the following (misconceptions):

**Table 5:** How likely is an IUD to cause the following? Correct answers are indicated by the "X".

	1	2	3	4	5
	Very Likely	Likely	Neither Likely nor Unlikely	Unlikely	Very Unlikely
Pelvic inflammatory disease				X	X
Spontaneous abortion (miscarriage)	X	X			
Get misplaced/fall out				X	X

21. Please choose if the following statements are true or false.

**Table 6:** Please choose if the following statements are true or false. Correct answers are indicated by the "X".

​​	True	False
A patient must have previously given birth to be eligible for an IUD.		X
IUD insertion is painful.	X	
IUDs can cause ectopic pregnancies.		X
IUDs negatively interfere with sexual pleasure.		X
IUDs can cause weight gain.		X
A patient must obtain a pap smear before receiving an IUD		X

22. Knowledge regarding IUDs.

**Table 7:** Knowledge regarding IUDs. Correct answers are indicated by the "X".

	Strongly Agree/Agree	Neither Agree nor Disagree	Disagree/ Strongly Disagree
Assuming she is an otherwise good candidate, I would recommend an IUD for a patient:			
Who has never been pregnant	X		
Who is under 19 years old	X		
Who currently has chlamydia			X
Who has had more than one vaginal delivery	X		

23. Attitudes regarding IUD counseling.

**Table 8:** Student attitudes regarding which clinicians should be able to COUNSEL patient about IUDs.

	1	2	3	4	5
	Strongly Agree	Agree	Neither Agree nor Disagree	Disagree	Strongly Disagree
The following clinicians should be able to COUNSEL patient about IUDs:					
Ob-Gyns					
Family Physicians					
Internal Medicine Physicians					
Pediatricians					
Midwives/Nurse Practitioners					

24. Attitudes regarding IUD placement.

**Table 9:** Student attitudes regarding which clinicians should be able to PLACE IUDs.

	1	2	3	4	5
	Strongly Agree	Agree	Neither Agree nor Disagree	Disagree	Strongly Disagree
The following clinicians should be able to PLACE IUDs:					
Pediatricians					
Midwives/Nurse Practitioners					
Internal Medicine Physicians					
Ob-Gyns					
Family Physicians					

25. Personal comfortability regarding IUDs

**Table 10:** Personal comfortability regarding IUDs.

	1	2	3	4	5
	Strongly Agree	Agree	Neither Agree nor Disagree	Disagree	Strongly Disagree
I am interested in learning more about IUDs					
I want to learn how to place IUDs					
Contraceptive counseling will be a part of my practice					
I would recommend an IUD to my family member					
I would get an IUD placed or I would recommend an IUD to my partner					

26. Self-efficacy regarding IUDs.

**Table 11:** Self-efficacy regarding IUDs.

	1	2	3	4	5
	Strongly Agree	Agree	Neither Agree nor Disagree	Disagree	Strongly Disagree
I feel confident that am able to counsel patient about the IUD					
I know the steps to place an IUD					
I feel comfortable placing an IUD independently in plastic model					
I could teach another student how to place an IUD in a plastic model					
I feel comfortable placing an IUD in a patient under faculty supervision					

END OF PRE-TEST

*With permission from the authors, portions of this survey have been used from a previously published manuscript (Bartz et al. [8]).

Post-Training Intrauterine Contraception Questionnaire

Please provide the last 4 digits of your cell phone (or home phone) number. _____________. This is used to pair your pre- and post- survey responses. It is not used to identify you in any way.

-Administer questions 16 through 26 in the pre-survey above.

END OF POST-TEST

*With permission from the authors, portions of this survey have been used from a previously published manuscript (Bartz et al. [8]).
